# Spatial variability in the density, distribution and vectorial capacity of anopheline species in a high transmission village (Equatorial Guinea)

**DOI:** 10.1186/1475-2875-5-21

**Published:** 2006-03-23

**Authors:** Jorge Cano, Miguel Ángel Descalzo, Marta Moreno, Zhaoguo Chen, Sisinio Nzambo, Leonardo Bobuakasi, Jesús N Buatiche, Melchor Ondo, Francisco Micha, Agustín Benito

**Affiliations:** 1 Centro Nacional de Medicina Tropical. Instituto de Salud Carlos III, C/Sinesio Delgado 6, Madrid, Spain; 2 Centro de Referencia para el Control de Endemias, Centro Nacional de Medicina Tropical, Instituto de Salud Carlos III, Equatorial Guinea; 3 Laboratorio de Malaria, Centro Nacional de Microbiología, Instituto de Salud Carlos III. Majadahonda, Madrid, Spain

## Abstract

**Background:**

Malaria transmission varies from one country to another and there are also local differences in time and space. An important variable when explaining the variability in transmission is the breeding behaviour of the different vector species and the availability of breeding sites. The aim of this study was to determine the geographical variability of certain entomological parameters: human biting rate (HBR), sporozoitic index (SI) for *Plasmodium falciparum *and entomological inoculation rate (EIR).

**Methods:**

The study was carried out in a small village in the mainland region of Equatorial Guinea. Adult mosquitoes were collected by CDC light traps. Polymerase Chain Reaction was employed to identify the species within the *Anopheles gambiae *complex and to detect *P. falciparum *sporozoites. The geographical position of all the dwellings in the village were taken using a global positioning system receiver unit. Data relating to the dwelling, occupants, use of bednets and the mosquitoes collection data were used to generate a geographical information system (GIS). This GIS allowed the minimum distance of the dwellings to the closest water point (potential breeding sites) to be determined.

**Results:**

A total of 1,173 anophelines were caught: 279 *A. gambiae s.l. *(217 *A. gambiae s.s. *and one *Anopheles melas*), 777 *Anopheles moucheti *and 117 *Anopheles carnevalei*. *A. moucheti *proved to be the main vector species and was responsible for 52.38 [95% IC: 33.7–71] night infective bites during this period. The highest SI was found in *A. carnevalei *(24%), even though the HBR was the lowest for this species. A significant association was found between the distance from the dwellings to the closest water point (River Ntem or secondary streams) and the total HBR.

**Conclusion:**

A clear association has been observed between the distance to potential breeding sites and the variability in the anopheline density, while the other parameters measured do not seem to condition this spatial variability. The application of GIS to the study of vector-transmitted diseases considerably improves the management of the information obtained from field surveys and facilitates the study of the distribution patterns of the vector species.

## Background

The transmission of malaria is intense in the majority of the countries of Sub-Saharan Africa, particularly in those that are located along the equatorial strip. In 2000, malaria was responsible for 18% (approx. 803,000) of deaths among children under five years of age in the African continent [1]. The criteria previously used to classify the malaria transmission level were based on parasitological and clinical data, splenic index and prevalence of the parasitaemia [2]. Nowadays, the entomological inoculation rate (EIR) is considered a key factor when establishing the degree of endemicity or transmission level [3]. Thus, an EIR under 1 is typical of a hypoendemic zone and a EIR between 100 and 1,000 identifies a holoendemic zone.

The EIR is the number of infective bites that an individual receives during a determined period of time. It is usually measured using the anophelines caught when they land on an individual who acts as bait [4-6]. This type of collection method has been widely discussed from ethical and technical point of views. Exposing technical staff to infective mosquitoes bites is ethically unacceptable, even when they are protected by a chemo-prophylactic treatment. On the other hand, differences in human attractiveness, motivation and diligence in the collection work give a certain degree of subjectivity to catching mosquitoes using human bait [7]. Using mechanized collection methods, such as light traps, solves the aforementioned problems, although there are discrepancies with respect to the quality of the information obtained and its application to determine the EIR. In Kenya, Mbogo *et al. *collected more mosquitoes by light traps than when using human bait [8] and Davis *et al. *detected a higher sporozoitic index (SI) in mosquitoes collected using CDC light traps, which is the opposite to what was observed by other authors [9]. Despite the discrepancies, the use of light traps placed near an individual protected by a non-impregnated mosquito net is becoming more generalized to determine the Human Biting Rate (HBR), particularly for endophagic mosquitoes [10-12].

Malaria transmission not only varies from one country to another, but there are also local differences in time and space [13]. Internal climatological differences are common in large African countries and even if they are not too marked, they may led to differences in the transmission pattern. Transmission differences associated to the degree of urban development can also be seen. Thus, malaria transmission is usually greater in rural areas than in urban areas, even though it is also relevant in peri-urban zones of certain African cities [14-16]. The variability in the transmission in a small village and the potential factors that determine that variability have been less studied [17]. The best known and most studied determining variability factors would include those relating to the different attractiveness of the potential hosts [18,19]. The emission of carbon dioxide [20], the production of lactic acid as the result of muscular metabolism [21] and other molecules, such as octenol [22] are known to be attracting factors.

Another highly important variable when explaining the variability in the transmission is the breeding behaviour of the different vector species and the availability of breeding sites. Numerous studies have described a negative correlation between distance from breeding points and anopheline density [23,24].

The aim of this paper was to determine the geographical variability of certain entomological parameters: HBR, SI for *Plasmodium falciparum *and EIR in a place with a high malaria transmission rate. The implication of certain factors (population, characteristics of the dwellings and distances from water points) on this spatial variability was also analysed.

## Methods

### Study location

**Figure 1:**
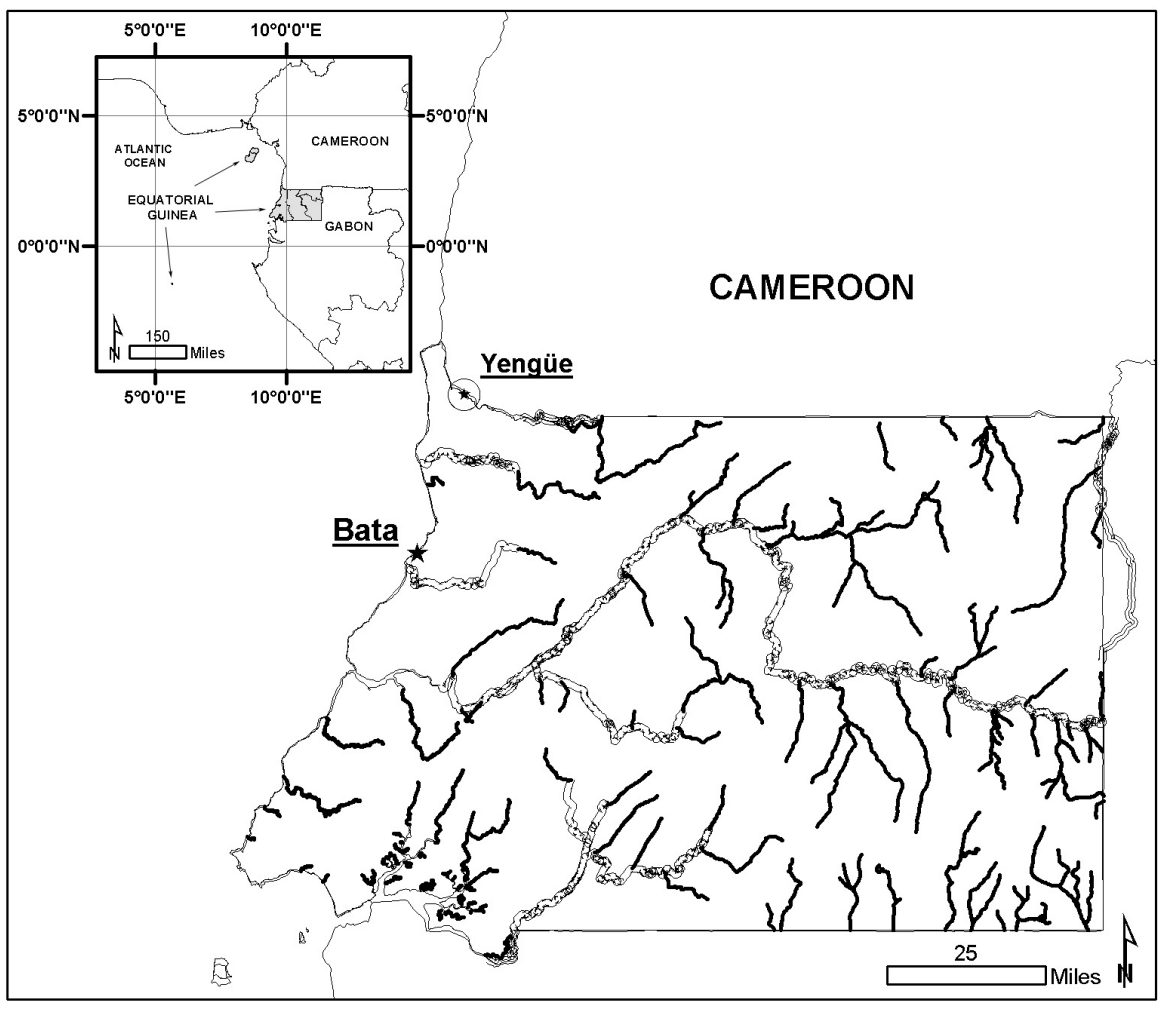
Map of the mainland region of the Equatorial Guinea and location of the Yengüe village.

Yengüe village (N 02° 13,392' E 009° 52,716') is located in the mainland region of Equatorial Guinea, in the Rio Campo municipality, in the frontier region with Cameroon (see Figure 1). The village is made up of three scattered units: Yengüe, Ncoho Mekah and Bilan. The village has a total of 37 dwellings, most of which are built out of wood with zinc roofs and earth floors.

The country's mainland region has a typically equatorial climate. There are four seasons: two dry seasons, one which lasts from December to the middle of March and the other from July to the middle of September, and two rainy seasons, a more intense one that lasts from the middle of September to the end of November and the other one from the middle of March until the end of June, when the rain is less intense. The annual rainfall amounts to 2,074 mm over 118 rainy days. The relative humidity ranges between 70% and 100% throughout the year. The average temperature is 25°C, with the minimum ranging between 17°C and 21°C and the maximum between 29°C and 30°C, depending on the location and the season [25].

### Collection methods

Adult mosquitoes were collected between April and June 2002, by means of seven normal light CDC traps (model 512, John Hock, Gainesville, FL). One trap was distributed per dwelling and positioned a metre and a half from the floor and close to the occupants who were protected as they slept using a non-impregnated bednet [26]. Two light traps were kept in the same dwellings throughout the 42-day collection period and, thus, acted as control or sentinel dwellings. One of the sentinel dwellings had more than five occupants and the other less than five. The other five light traps were rotated through the rest of the dwellings that made up the village, in such a way that each dwelling was surveyed between 6 and 8 times during the collection period. If one of the selected dwellings was closed or empty on the day it was to be surveyed, the study was carried out in the immediately posterior dwelling. If that was also closed, the study was carried out in the previous one. The light traps were switched on each day at 18.00 hours and were collected the following morning (06.00 hours).

The captured mosquitoes were transported to the reference laboratory in cardboard containers in order to be subsequently identified and stored in individual tubes with silica-gel.

### Identifying and processing the mosquitoes

All mosquitoes were frozen, separated into Culicinae and Anophelinae and counted. The collected anophelines were identified using the keys described by Gillies and De Mellion [27], Gillies and Coetzee [28] and Hervy *et al. *[29], recording sex and feeding state, and finally stored in silica-gel for subsequent molecular studies.

Head and thorax of the females conserved in silica-gel were lysed and the DNA extracted using the "Bender Buffer Lysis" method. DNA was used to perform a PCR to identify the species within the *Anopheles gambiae *complex and another PCR to identify infective specimens (*P. falciparum *sporozoites presents in head-thorax).

PCR for species identification was performed using a slightly modified version (in the "master mix" and the amplification programme times) of the protocol described by Scott *et al *[30]. DNA of *Anopheles melas*, *A. gambiae s.s.*, *Anopheles arabiensis*, *Anopheles quadriannulatus *(supplied by Prof. Dr. Virgilio do Rosario from the Centro de Malária e Outras Doenças Tropicáis, Lisboa, Portugal) was used as positive controls. Sterilized water was used as the negative control.

PCR to detect *P. falciparum *sporozoites was designed using the fragment of 753 base pair insert of pBRK1–14 as described by Fucharoen *et al *[31]. DNA amplification was performed according to the protocol described by Tassanakajon *et al *[32]. A band of 206 base pairs was obtained in positive samples. Control *P. falciparum *DNA was obtained from adult female experimentally infected from a colony of *Anopheles stephensi *kept at the Centro Nacional de Medicina Tropical (Madrid, Spain).

### Geographical and statistical analysis

The longitude and latitude data of all the dwellings in the village were taken using a global positioning system (GPS) receiver unit. Data relating to the dwelling (type of construction, floor and roof), number of occupants and children under five years of age, use of bednets and the data relating to the anopheline gathered (total mosquitoes captured per species, HBR, SI, and EIR) were used to generate a geographical information system (GIS) and, thus, be able to study the spatial association of the entomological variables with the rest of the factors. This GIS allows the minimum distance (Euclidean distance) of the dwellings to the closest water point (River Ntem or secondary stream) to be determined. This data was introduced in the analysis as another variable to be taken into account.

The HBR was calculated using the light trap collections. Given that more than one person could be occupying a dwelling, and having distributed one trap per house, we consider that it is more accurate to refer to the HBR obtained by dwelling and not by person. The EIR would likewise be calculated by dwelling.

Using the aforementioned study variables, a multivariate linear regression analysis was performed to estimate the association of these variables with overall HBR and by species, and with overall SI. P-values <0.05 were considered to be statistically significant.

## Results

A total of 1,173 anophelines were caught during 42 collection nights between April and June 2002: 279 (23.8%) *A. gambiae s.l.*, 777 (66.2%) *A. moucheti *and 117 (10%) *A. carnevalei*. PCR for species identification was performed on 218 (78%) mosquitoes belonging to the gambiae complex, which results in 217 *A. gambiae s.s. *and one *A. melas*.

**Figure 2:**
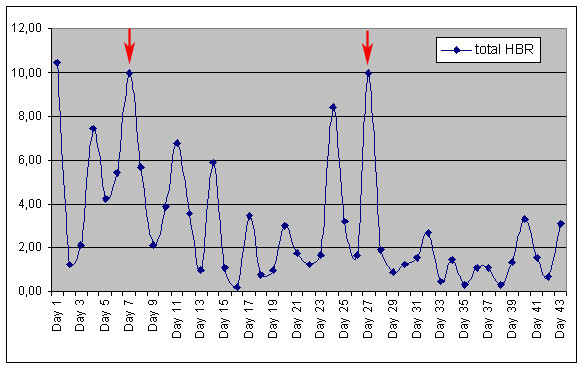
Evolution of the total Human Biting Rate.

EIR for the survey period (rainy season) was determined by multiplying HBR by SI. *A. moucheti *proved to be the main vector species and was responsible for 52.38 [95% IC: 33.7–71] night infective bites during this period. The secondary vectors in the village were *A. gambiae s.s. *and *A. carnevalei*, in that order. The highest SI was found in *A. carnevalei *(24%), although the HBR was the lowest for this species (Table 1). Figure 2 represents HBR evolution followed for the three species throughout the study. Two marked peaks were observed between days 7 and 9 and days 25 and 27, which coincide with periods of maximum hatching. This time interval coincides with the average duration of the larval cycle (14–16 days).

**Table 1:** HBR, SI, EIR per night and EIR per season

		**HBR**	**SI**	**EIR_night**	**EIR_season**
***A. gambiae *s.s.**	279	1.09 [0.76–1.41]	0.15 [0.075–0.224]	0.16 [0.086–0.238]	14.77 [7.846–21.696]
***A. moucheti***	777	3.07 [1.72–4.41]	0.19 [0.144–0.232]	0.58 [0.371–0.781]	52.38 [33.718–71.034]
***A.carnevalei***	117	0.45 [0.03–0.86]	0.24 [0.191–0.294]	0.11 [0.040–0.178]	9.90 [3.604–16.187]
**Total**	**1173**	**4.60 [2.71–6.49]**	**0.18 [0.140–0.230]**	**0.85 [0.354–1.349]**	**77.47 [32.205–122.732]**

**Figure 3:**
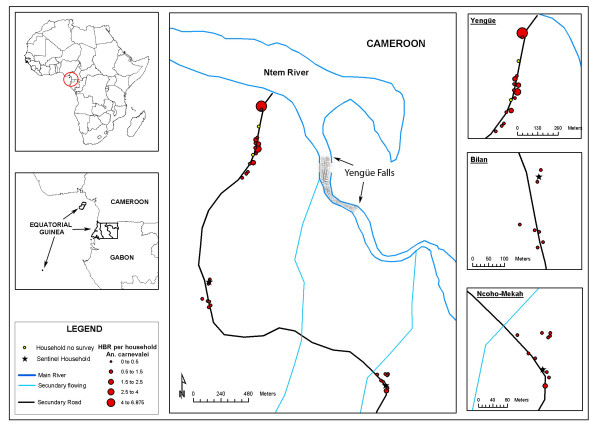
Human Biting Rate for *Anopheles carnevalei *according to dwelling.

**Figure 4:**
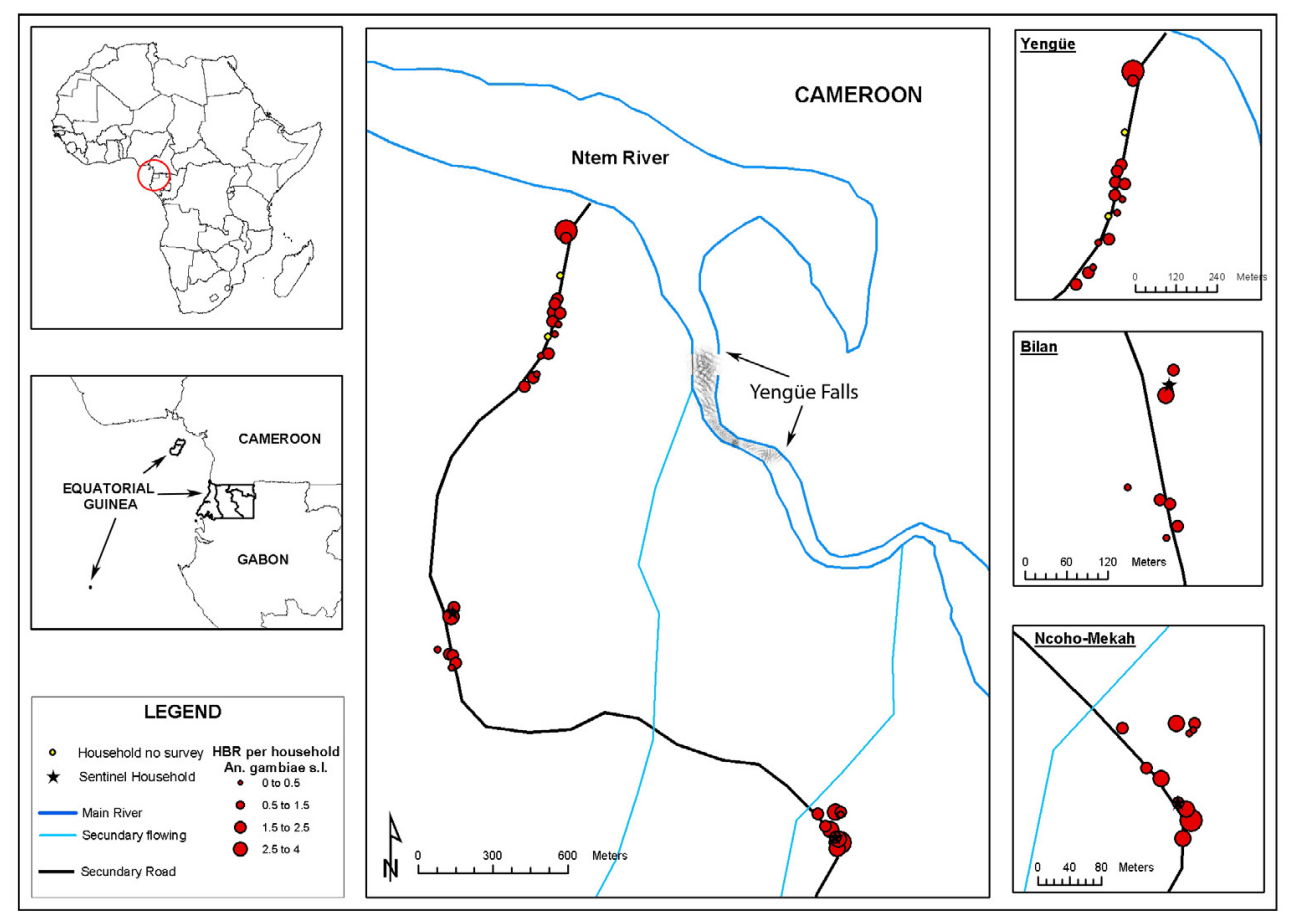
Human Biting Rate for *Anopheles gambiae s.l *according to dwelling.

**Figure 5:**
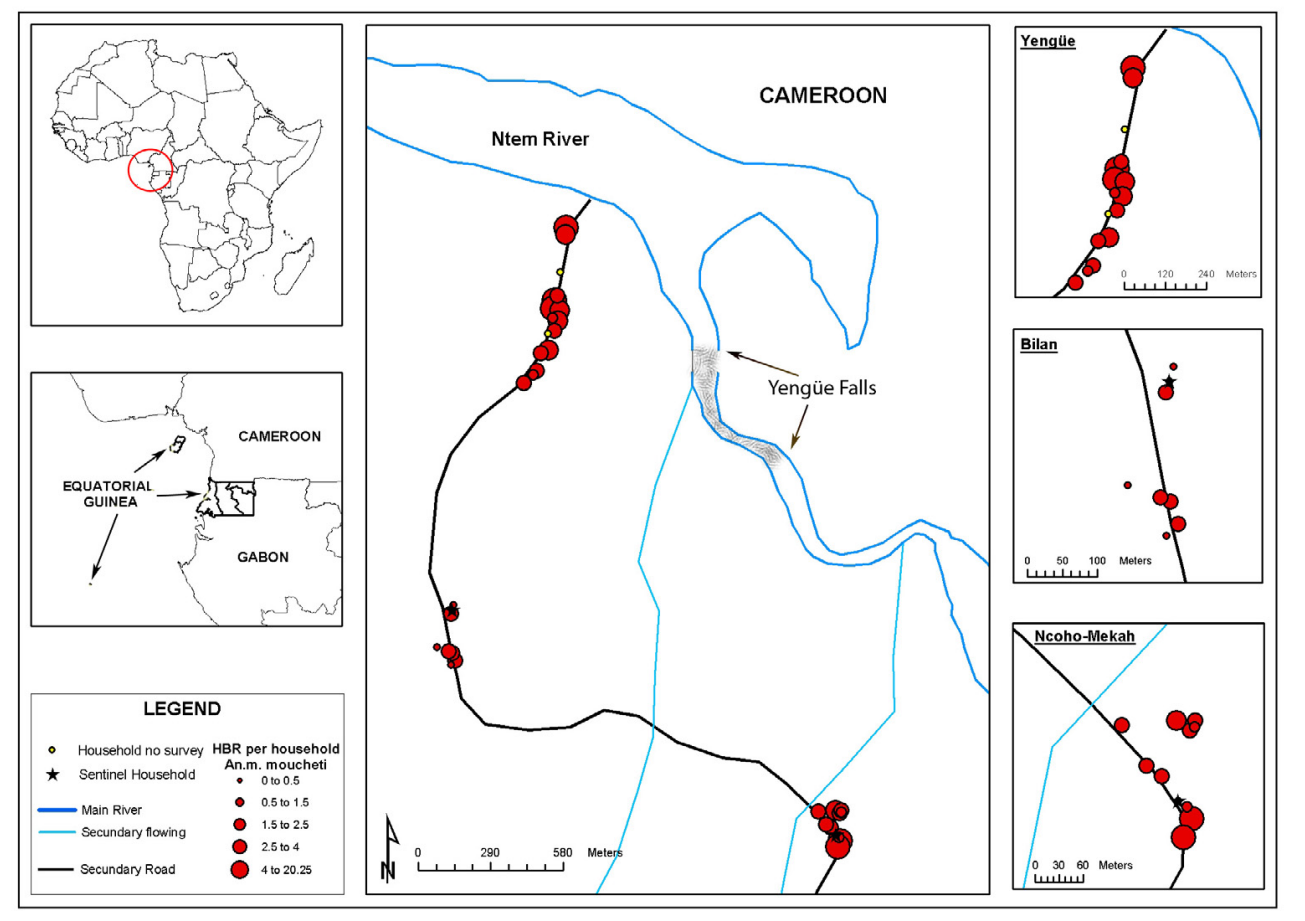
Human Biting Rate for *Anopheles moucheti *according to dwelling.

The association of the different study variables was assessed with the HBR and the SI of the three vector species. The HBR and SI values needed to be transformed for the analysis (neperian logarithm). A significant association was found between the distance from the dwellings to the closest water point (River Ntem or secondary streams) and the total HBR (Table 2). At species level, an association was only noted between the distance to the water and the HBR for *A. moucheti *(coefficient: -0.002, p = 0.007). No association was found between the variables studied and the SI. The analysis of outliers and influential observations showed three different zones that each tallied with one of the three geographical units that make up the village (Figures 3, 4, 5). From then onwards, the multivariate analysis was reformulated and each unit was considered independently. Yengüe (zone 1), Bilan (zone 2) and Ncoho-Mekah (zone 3).

**Table 2:** Potential predicting factors of the total HBR and SI

				**Ln total HBR**		**Ln total SI**	
**Variable**	**Type**	**n (%)**	**Average**	**Coeff.**	**p-value**	**Coeff.**	**p-value**

**Type of building**	**Cement**	1 (2.9%)					
	**Wood**	31 (88.6%)		1.014 [-0.04–2.06]	0.059	0.143 [-0.21–0.49]	0.410
	**Mud**	3 (8.6%)		1.104 [-0.59–2.80]	0.193	0.213 [-0.19–0.61]	0.283
**Roof**	**Sheeting**	23 (65.7%)					
	**Nipas**	12 (34.3%)		0.494 [-0.24–1.23]	-0.064	-0.064 [-0.17–0.04]	0.235
**Open Roof**	**Yes**	35 (100%)					
	**Not**	0					
**Floor**	**Cement**	7 (20%)					
	**Earth**	28 (80%)		0.138 [-0.70–0.97]	0.736	0.060 [-0.08–0.20]	0.392
**Bednet**	**Yes**	11 (31.4%)					
	**No**	24 (68.6%)		0.109 [-0.58–0.80]	0.748	-0.081 [-0.19–0.03]	0.142
**No. bednets**		12	0.34 [0.138–0.542]				
**Occupants**		189	5.4 [4.308–6.492]	0.054 [-0.117–0.225]	0.522	-0.015 [-0.04–0.01]	0.215
**Under 5s**		44	1.26 [0.812–1.708]	0.139 [-0.279–0.556]	0.500	0.026 [-0.04–0.09]	0.434
Distance to river*			429.6 [335.02–524.17]	-0.002 [-0.003–0.000]	**0.008**	0.000 [0.00–0.00]	0.609

*p <0.05

Most of the dwellings in zone 1 were located near to the River Ntem (potential breeding places) while dwellings of zone 2 and 3 were located closes to the secondary streams. Based on this, the GIS was used to provide the distance from all the dwellings in the three villages to an area of rapids along the River Ntem, where ponds and pools are frequently formed. A significant association was only found between zone 1 and the overall HBR (coefficient: -0.003, p = 0.017) and by species: *A. moucheti *(coefficient:-0.004, p = 0.003) and *A. carnevalei *(coefficient:-0.001, p = 0.017).

## Discussion

The transmission of malaria varies within a location and is dependent on time and space variables. During this study, conducted in a small village, near to a large river and where three anopheline species live with markedly endophagic and antropophagic behaviour, the importance that the availability of suitable breeding places and their distance from the dwellings has on the HBR has been made clear.

The data obtained in this study agree with what has been observed in neighbouring countries, such as Cameroon, where *A. moucheti *is the main vector species in the rural areas and is particularly associated with river beds [33]. *A. moucheti *usually breeds in slow flowing rivers where there are small islands with vegetation made up of species such as *Pistia stratiotes *and *Paspallum spp. *[27], while *A. gambiae s.s. *shows a clear preference for temporary pools, which are often the result of human activity [34,35]. The characteristic intense rain of the period when the study was conducted may hinder the breeding of anopheline species, which, such as *A. gambiae s.s*., show a clear preference for small pools (tracks, footprints, etc...) *A. carnevalei *is a recently described species based on specimens from collections coming from the Ivory Coast and Cameroon. They were initially identified as a "clear form" of *Anopheles nili*. More detailed taxonomic studies lead to its description as a different species [36]. In recent years, its presence has been described in the mainland area of Equatorial Guinea, in areas close to rapids, as is the case of Yengüe [37]. Probably, in the same way as *A. nili, A. carnevalei *chooses the surrounding vegetation of the small pools or lakes that form on the banks of rivers and waterfalls as its breeding ground. As far as the vectorial capacity is concerned, *A. carnevalei *is highly receptive to *P. falciparum *infection and has the highest infection percentage of the three species. The fact that it has a lower HBR means that it is classified as a secondary vector in the study area. This is the first study where the role that this species plays in malaria transmission has been described in detail.

In any case, that surveys need to be conducted at other periods of the year (dry season) to determine the role that each of these species plays in malaria transmission.

Over the last 20 years, the technology for studying spatial or geographical phenomena has advanced considerably: the global positioning systems have been improved, space satellites with high resolution remote sensors launched, geographical information systems (GIS) developed... However, the application of this technology to the study of epidemiology is relatively recent and its development is still at a very early stage [38]. Given that the spatial distribution of vector-transmitted diseases is defined by the geographical distribution of the vectors and their vertebrate hosts, the epidemiological surveillance and control programmes should include spatial management of the different transmission parameters. In this case, the GIS based on the field information not only has provided a graphical representation of the spatial variability in the density of the three vector population, but has also allowed to generate a new variable, the distance to potential breeding grounds. Out of all the variables included in the study, this has been the only one that has shown a clear association with the spatial variability in the HBR.

The different attractiveness of each host can be expected to condition the number of bites that an individual receives on average per night compared to other people in the same dwelling, but it does not have such an influence on the indoor mosquito density. During a study conducted in Ifakara, Tanzania, the density of anophelines per dwelling was seen to have a negative correlation with the distance to the breeding points [35]. Similar results have been observed in different ecological conditions [39].

In conclusion, a clear association has been observed in the study zone between the distance to potential breeding places and the variability in the anopheline density, while other parameters such as the number of occupants, children per house, or type of construction, do not seem to condition this spatial variability. On the other hand, the application of the GIS to the study of vector-transmitted diseases considerably improves the management of the information obtained from the field surveys and facilitates the study of the distribution patterns of the vector species.

## Authors' contributions

JC was involved in the design of the survey, performed the GIS model, participated in the data collection and the interpretation of statistical analysis, and coordinated the draft of the manuscript. MD was performed the statistical analysis and interpretation and drafted the manuscript. MM was involved in the molecular studies and helped to draft the manuscript. ZC was involved in the molecular studies. SN, LB, JB, MO and FM participated in the collection of the data and mosquitoes identification. AB participated in the design of the surveys and have given approval of the version to be published. All authors read and approved the final manuscript.

## References

[B1] WHO/UNICEF/RBM (2005). World Malaria Report 2005.

[B2] Gilles HM, Warrel DA, Edward Arnold (1993). Epidemiology of malaria. Bruce-Chwatt's essential malariology.

[B3] Beier JC, Killeen GF, Githure J (1999). Short report: entomologic inoculation rates and *Plasmodiumfalciparum *malaria prevalence in Africa. Am J Trop Med Hyg.

[B4] WHO (1975). Manual on practical entomology in malaria.

[B5] Antonio-Nkondjo C, Awono-Ambene P, Toto JC, Meunier JY, Zebaze-Kemleu S, Nyambam R, Wondji CS, Tchuinkam T, Fontenille D (2002). High malaria transmission intensity in a village close to Yaounde, the capital city of Cameroon. J Med Entomol.

[B6] Killeen GF, McKenzie FE, Foy BD, Schieffelin C, Billingsley PF, Beier JC (2000). The potential impact of integrated malaria transmission control on entomologic inoculation rate in highly endemic areas. Am J Trop Med Hyg.

[B7] Lindsay SW, Adiamah JH, Miller JE, Pleass RJ, Armstrong JRM (1993). Variation in attractiveness of human subjects to malaria mosquitoes (Diptera: Culicidae) in the Gambia. J Med Entomol.

[B8] Mbogo CNM, Glass GE, Forster D, Kabiru EW, Githure JI, Ouma JH, Beier JC (1993). Evaluation of light traps for sampling Anopheline mosquitoes in Kilifi, Kenya. J Am Mosq Control Assoc.

[B9] Davis JR, Hall T, Chee EM, Majala A, Minjas J, Shiff CJ (1995). Comparison of sampling anopheline mosquitoes by light-trap and human-bait collections indoors at Bagamoyo, Tanzania. Med Vet Entomol.

[B10] Aranda C, Aponte JJ, Saute F, Casimiro S, Pinto J, Sousa C, Do Rosario V, Petrarca V, Dgedge M, Alonso P (2005). Entomological characteristics of malaria transmisión in Manhiça, a rural area in Southern Mozambique. J Med Entomol.

[B11] Shiff CJ, Minjas JN, Hall T, Hunt RH, Lyimo S, Davis JR (1995). Malaria infection potential of anopheline mosquitoes sampled by light trapping indoors in coastal Tanzanian villages. Med Vet Entomol.

[B12] Ijumba JN, Mosha FW, Lindsay SW (2002). Malaria transmission risk variations derived from different agricultural practices in an irrigated of northern Tanzania. Med Vet Entomol.

[B13] Mapping Malaria Risk in Africa. [http://www.mara.org.za].

[B14] Robert V, Macintyre K, Keating J, Trape JF, Duchemin JB, Warren M, Beier JC (2003). Malaria transmission in urban sub-Saharan Africa. Am J Trop Med Hyg.

[B15] Keating J, Macintyre K, Mbogo C, Githeko A, Regens JL, Swalm C, Ndenga B, Steinberg LJ, Kibe L, Githure JI, Beier JC (2003). A geographic sampling strategy for studying relationships between human activity and malaria vectors in urban Africa. Am J Trop Med Hyg.

[B16] Sagara I, Sangare D, Dolo G, Guindo A, Sissoko M, Sogoba M, Niambele MB, Yalcoue D, Kaslow DC, Dicko A, Ilion AD, Diallo D, Millar LH, Toure Y, Doumbo O (2002). A high malaria reinfection rate in children and young adults living under a low entomological inoculation rate in a periurban area of Bamako, Mali. Am J Trop Med Hyg.

[B17] Fontenille D, Lochouarn L, Diagne N, Sokhna C, Lemasson JJ, Diatta M, Konate L, Faye F, Rogier C, Trape JF (1997). High annual and seasonal variations in malaria transmission by anophelines and vector species composition in Dielmo, a holoendemic area in Senegal. Am J Trop Med Hyg.

[B18] Enserink M (2002). What mosquitoes want: secrets of host attraction. Science.

[B19] Knols BGJ, de Jong R, Takken W (1995). Differential attractiveness of isolated humans to mosquitoes in Tanzania. Trans R Soc Trop Med Hyg.

[B20] Constantani C, Gibson G, Sagnon N, Della Torre A, Brady J, Coluzzi M (1996). Mosquito response to carbon dioxide in a West African Sudan savanna village. Med Vet Entomol.

[B21] Geier M, Sass H, Boeckh J (1996). A search for components in human body odour that attract females of Aedes aegypti. Ciba Found Symp.

[B22] Kline DL (1994). Olfactory attractants for mosquito surveillance and control: 1-octen-3-ol. J Am Mosq Control Assoc.

[B23] Minakawa N, Seda P, Yan G (2002). Influence of host and larval habitat distribution on the abundante of african malaria vectors in Western Kenya. Am J Trop Med Hyg.

[B24] Minakawa N, Sonye G, Mogi M, Yan G (2004). Habitatcharacteristics of *Anopheles gambiae s.s. *larvae in a Kenyanhighland. Med Vet Entomol.

[B25] Alvar J, Mas-Coma S, Carrasco M (1996). Modern history and physical geography of Equatorial Guinea. Research and Reviews in Parasitology.

[B26] Mboera LFG, Kihonda J, Braks MAH, Knols BGJ (1998). Influence of Centres for Disease Control light trap position, relative to a human-baited bed net, on catches of *Anopheles gambiae *and *Culex quinquefasciatus *in Tanzania. Am J Trop Med Hyg.

[B27] Gillies MT, De Mellion B (1968). The Anophelinae of Africa south of the Sahara (Ethiopian zoogeographical region). the South African Institute for Medical Research.

[B28] Gillies MT, Coetzee MT (1987). A Supplement to the Anophelinae of Africa south of the Sahara (Ethiopian zoogeographical region). the South African Institute for Medical Research.

[B29] Hervy JP, Le Goff G, Geoffroy JP, Hervé L, Manga L, Brunhes J (1998). Les anopheles de la région Afro-tropicale. Logiciel d'identification et d'enseignement. ORSTOM édition série Didactiques Paris, France (in French, English, Portuguese).

[B30] Scott JA, Williams G, Collins FH (1993). Identification of single specimens of the *Anopheles gambiae *complex by the polymerase chain reaction. Am J Trop Med Hyg.

[B31] Fucharoen D, Tirawanchai N, Wilairat P, Panyim S, Thaithong S (1988). Differentiation of Plasmodium falciparum clones by means of a repetitive DNA probe. Trans R Soc Trop Med Hyg.

[B32] Tassanakajon A, Boonsaeng V, Wilairat P, Panyim S (1993). Polymerase chain reaction detection of *Plasmodium falciparum *in mosquitoes. Trans R Soc Trop Med Hyg.

[B33] Antonio-Nkondjio C, Simard F, Awono-Ambene P, Ngassam P, Toto JC, Tchuinkam T, Fontenille D (2005). Malaria vectors and urbanization in the equatorial forest region of south Cameroon. Trans R Soc Trop Med Hyg.

[B34] Manga L, Fondjo E, Carnevale P, Robert V (1993). Importance of low dispersion of *Anopheles gambiae *(Diptera: Culicidae) on malaria transmission in hilly towns in south Cameroon. J Med Entomol.

[B35] Charlwood JD, Kihonda J, Sama S, Billingsley PF, Hadji H, Verhave JP, Lyimo E, Luttikhuizen PC, Smith T (1995). The rise and fall of *Anopheles arabiensis *(Diptera: Culicidae) in a Tanzanian village. Bull Entomol Res.

[B36] Brunhes J, Le Goff G, Geoffroy B (1999). Afro-tropical anopheline mosquitoes III. Description of three new species: *Anopheles carnevalei sp. nov, An. hervyi sp. nov*., and resurrection of *An. rageaui *Mattingly and Adam. J Am Mosq Control Assoc.

[B37] Cano J, Nzambo S, Buatiche JN, Ondo-Esono M, Micha F, Benito A (2003). *Anopheles *(Cellia) *carnevalei *in Equatorial Guinea (West-Central Africa). J Am Mosq Assoc.

[B38] Hay SI, Snow RW, Rogers DJ (1998). Form predicting mosquito habitat to malaria seasons using remotely sensed data; practice, problems and perspectives. Parasitol Today.

[B39] Minakawa N, Seda P, Yan G (2002). Influence of host and larval habitat distribution on the abundante of african malaria vectors in western Kenya. Am J Trop Med Hyg.

